# New Drugs, Appliances, and Things Medical

**Published:** 1892-01-09

**Authors:** 


### NEW DRUGS, APPLIANCES, AND THINGS MEDICAL.

[All preparations' appliances, novelties, etc., of which a notice is desired, should be sent for The Editor, to care of The Manager, 140, Strand, London, W.C.]

### THE CLAXTON COMBINED BED TABLE, AND BOOK RACK.

This is a most ingenious and useful invention. It consists of a table for invalids who are unable to sit up in bed, and a book-rack which can be lowered and raised at will to a position which will enable the invalid to read either lying down or sitting up. The special feature of this invention is that the table is left quite free whether the book-rack is in use or not. The table is about one foot in height by 22½ inches long and 10½ inches wide. At each end of the table cloth pockets are placed, to contain a book or other things in use. The table easily folds up into a very small space, which makes it most convenient to use when travelling. The inventor is Mrs. Claxton, Dovecote, New Brighton, Cheshire, and a specimen of the table may be seen at THE HOSPITAL Office, 140, Strand, W.C. It is made in polished mahogany and stained pine, the price varying from two guineas to 16s. 6d. Amongst the many appliances for the sick-room this will be found by far the most useful and compact.

### BOVRIL AND ITS PREPARATIONS.

Sometime back we published a note of the chemical investigation of bovril. We then stated that it was an excellent food and stimulant, and that the results of our examination were most satisfactory. The company manufacturing this indispensable household requirement have recently forwarded to us samples of the parent product and its offshoots.

First, then, what is bovril? It is a combination of the best extract of meat, with the powder of dried beef. That is, it is not only a stimulant (extract), but an actual food (dried beef), the more valuable as the stimulating powers of the extract enable the feeding powers of the dried beef powder to be quicker made use of than if the powder were taken alone. In our former notice we drew attention to what is a very well-known fact, viz., the actual uselessness of meat extract, or even the best beef tea, as a food. But it is a well-known clinical fact that the stimulating powers of a little good beef tea or extract will often enable the patient to take and digest other forms of refreshment which have a solid food value. Bovril ingeniously combines these ideas, and forms at once food and stimulant.

The company have combined their manufacture in various ingenious and tasty ways. First in our estimation come the soup powders. These are of various kinds, such as mulligatawny, kidney, &c., &c. The soup powder is put up in little tins, sufficient for a large plate of soup. Full directions are given, and no one can desire a pleasanter food. Then there are bovril chocolate and bovril cocoa, both most nutritious and agreeable to the palate. Bovril meat lozenges contain in a small compass a large amount of restorative material, particularly useful to travellers who have far to go between meals, as they occupy little space, and really keep one going. Another valuable combination is bovril wine. We have tried this lately in a case of great exhaustion during a prolonged attack of septicæmia. The results have been very satisfactory.

Besides the bovril itself, which we have described as meat extract, plus beef powder, the company also manufacture the extract separately; this is is the same as Liebig's and other forms of extract of meat. It serves admirably as a gravy maker or a gentle liquid stimulant, but has no food value *per se.* We do not know if the company sells the powdered beef alone, but we can imagine that it would be of great use both in the kitchen and sick-room. Finally, in answer to the question—From what is bovril extract and meat powder made?—we reply the best oxen in South America. Fed on the grass of the wide plains of the cattle country, the animals have the best chance of keeping healthy, and consequently their extract and beef powder of being the most wholesome meat derivatives to be met with.

